# Analysis of the concentrations and size distributions of cell-free DNA in schizophrenia using fluorescence correlation spectroscopy

**DOI:** 10.1038/s41398-018-0153-3

**Published:** 2018-05-22

**Authors:** Jie Jiang, Xueli Chen, Liya Sun, Ying Qing, Xuhan Yang, Xiaowen Hu, Chao Yang, Tianle Xu, Jijun Wang, Peng Wang, Lin He, Chaoqing Dong, Chunling Wan

**Affiliations:** 1 Bio-X Institutes, Key Laboratory for the Genetics of Developmental and Neuropsychiatric Disorders (Ministry of Education), Shanghai Key Laboratory of Psychotic Disorders, Shanghai Mental Health Center, Shanghai Jiao Tong University, Shanghai, 200030 China; 2 College of life sciences, Anhui Normal University, Wuhu, 241000 China; 3 Discipline of Neuroscience, Department of Anatomy, Histology and Embryology, Collaborative Innovation Center for Brain Science, Shanghai Jiao Tong University School of Medicine, Shanghai, 200025 China; 4 Collaborative Innovation Center of Genetics and Development, Shanghai, 200025 China; 5 The Fourth People’s Hospital of Wuhu, Wuhu, 241002 China; 6 School of Chemistry and Chemical Engineering, Shanghai Jiao Tong University, Shanghai, 200240 China

## Abstract

Cell-free DNA (cfDNA), which is primarily released following cell death, has been described and developed to serve as an effective biomarker in autoimmune diseases which may share the pathogenesis with schizophrenia. In this study, we hypothesized and explored whether the concentrations and size distributions of cfDNA are abnormal in schizophrenia. A total of 65 patients with schizophrenia (SZ), 29 patients with mood disorders (MD) and 62 matched healthy controls (HC) were included in the study. Fluorescence correlation spectroscopy was used to assay the molar concentrations and size distributions of cfDNA. Fluorometric quantification and quantitative real-time PCR (qPCR) were performed to verify the results. The cfDNA levels were approximately two-fold higher in the SZ group ((29 ± 15) nM) than in the healthy controls ((15 ± 9) nM; *P*-value = 0.00062), but the levels in patients with MD were not significantly different from those in the healthy controls ((17 ± 10) nM; *P*-value = 0.343). According to the size distribution analysis, cfDNA in schizophrenia patients was composed of shorter DNA molecules and showed an apoptosis-like distribution pattern. Our study shows the elevated levels and short sizes of cfDNA in schizophrenia patients, which provide direct evidences supporting increased apoptotic activity in the disease. cfDNA may be developed to serve as an auxiliary diagnostic marker for the disease in the future.

## Introduction

Schizophrenia is a devastating and poorly understood neuropsychiatric syndrome that affects ~1% of the world’s population^1^. Although several etiological hypotheses have been proposed for schizophrenia, including neurotransmitter abnormalities, developmental or neurodegenerative processes and immune system disturbance^2^, none of these hypotheses completely explains the complex symptoms that are seen in different patients. Due to our limited understanding of the origin and mechanism by which the disease develops, we have not yet taken full advantage of using these changes as potential diagnostic and therapeutic targets.

Apoptosis, which leads to the release of nucleic acids, is a physiological process of cell death that has been proven to be involved in the pathological process of schizophrenia. First of all, apoptosis plays important roles in the control of immune responses^3^ that has been proven to be abnormal in schizophrenia. Aside from its roles in immunity, the apoptotic process is highly active during neurodevelopment, including eliminating abnormal neurons during fetal brain development and throughout an individual’s lifespan^4^. Early research has estimated that 20–80% of all neurons in the central nervous system (CNS) die by apoptosis^5^. Studies have indicated activated apoptosis in schizophrenia by detecting proteins associated with apoptosis, analyzing single-nucleotide polymorphisms, and measuring caspase-3 activity in both the brain and peripheral blood^4,6–9^.

cfDNA was first observed by Mandel and Metais in autoimmune disease in 1948^10^ which may share the pathogenesis with schizophrenia. After that, cfDNA has since been described in various diseases in which cell death is thought to have a pathogenic role, including tumors^11,12^, trauma, stroke, myocardial infarction, and sepsis, as well as in physiological conditions such as pregnancy^13–17^. It has been proven by gel electrophoresis, electron microscope, and paired-end sequencing that the most abundant cfDNA fragments in both patients and healthy people were ~180 bp in length, which is a size characteristic of DNA released from cells undergoing apoptosis^12,18^. Quantification and size distribution analysis of cfDNA in different conditions has established numerous new possibilities for both researches into disease mechanisms and biomarker studies^18^. As noted above, dysfunctional apoptosis is potentially involved in the pathogenesis of schizophrenia, and apoptosis is the major physiological process that results in the release of cfDNA. Based on this information, we speculated that patients with schizophrenia would have increased levels of cfDNA and that their cfDNA would show a disease-specific size distribution pattern.

qPCR and paired-end sequencing are two techniques that are widely used to investigate cfDNA concentrations and size distributions in different diseases. However, qPCR is only applicable to preselected loci, and paired-end sequencing is time consuming and costly. Fluorescence correlation spectroscopy (FCS) is a technique for single-molecule detection that analyzes the fluctuations in fluorescence resulting from the Brownian motion of fluorescent molecules in a very small detection volume^19–21^. Previous study using FCS has demonstrated DNA molecules of different sizes released from cells following drug-induced apoptosis^22^. As noted in our previous study, a maximum entropy method (MEM)-based fitting procedure to analyze FCS raw data, called MEMFCS^23^, was successfully used to investigate the size distributions of DNA molecules in the multiple-component model. Therefore, FCS can be used to sensitively, selectively, and rapidly measure the concentration and fragmentation of DNA without requiring DNA separation.

## Materials and methods

### Subjects

A total of 68 patients with schizophrenia (the SZ group), 34 patients with mood disorders (the MD group), and 65 matched healthy controls (the HC group) were recruited in Anhui Province, China, following the guidelines of the local ethics committee. Patients were experiencing their first-onset psychosis or had relapsed after at least 1 year without any antipsychotic drugs. The diagnosis of schizophrenia and mood disorders was based on the criteria of the Diagnostic and Statistical Manual of Mental Disorders, Fourth Edition. The study was approved by the Ethics Committee of the Bio-X Institutes, Shanghai Jiao Tong University.

### Plasma collection and cfDNA isolation

In total 5 ml of EDTA-anticoagulated peripheral blood were drawn from each subject at the time of recruitment. Plasma was obtained by centrifugation of fresh blood at room temperature and 1600 × *g* for 10 min followed by a second step of centrifugation at 4 °C and 16,000 × *g* for 10 min to remove cellular debris. The supernatants were stored at −80 °C. cfDNA was extracted from 0.6 mL of plasma and dissolved in 30 μL ddH_2_O using the TIANamp Micro DNA Kit (TIANGEN, China) according to the manufacturer’s protocol.

### Preparation of DNA base pairs with known length

Three DNA base pairs with length of 180 bp, 360 bp, and 540 bp were used as standards for size determination. All three base pairs were prepared by PCR and purified from agarose gels using QIAquick Gel Extraction Kit (Qiagen, German) according to the manufacturer’s protocol. The primers for each pair are presented in supplementary Table S2.

### DNA detecting by FCS

cfDNA extracted from plasma and DNA base pairs prepared by PCR were measured with a FCS system built in-house. SYBR Green I (10,000 × concentrate in DMSO) purchased from Molecular Probes Inc. (S7563, 796325, USA) was used as a dye to combine dsDNA for FCS detection. 8 nM Rhodamine Green was used as a reference dye for instrument calibration and the detection volume size was determined to be about 0.5 fL (the diffusion time of Rhodamine Green is measured as 54.3 ± 0.5 μs and the ratio of the axial and lateral radius of the volume (z0/ω0) is 6.1). FCS measurements were performed over a period of 240 s in a single run at room temperature and were repeated three times. Details of experimental procedures and data processing could be found in supplementary information.

### Fluorometric quantification and qPCR

To verify the results obtained by FCS, we performed experiments using two different methods: absolute quantification using a fluorometer and relative quantification using qPCR. Total cfDNA was measured using the Quant-iT^TM^ dsDNA High-Sensitivity Assay Kit and a QubitVR 2.0 Fluorometer (Invitrogen, CA) following the manufacturer’s instructions. The targets for qPCR were two consensus sequences of human ALU-interspersed repeats: a 115 bp ALU amplicon (ALU115) that represents both shorter and longer cfDNA fragments, and a 247 bp ALU amplicon (ALU247) that represents only longer DNA fragments. The sequences of the primers were shown in supplementary Table S2. A 4.8 µL diluted cfDNA template was used with 1 × SYBR Green master mix (Roche, Switzerland) for qPCR, which was performed by a ViiA™ 7 Real-Time PCR System (Life Technologies, USA) at 95 °C for 10 min, followed 35 cycles of denaturation at 95 °C for 30 s, annealing at 64 °C for 30 s, and extension at 72 °C for 30 s^13^. All qPCR assays were performed in a blinded fashion without knowledge of the specimen identity, and mean values were calculated from triplicate reactions.

### Statistical analysis

Logistic regression analysis was conducted for group comparisons and to adjust for confounding factors including age, gender, BMI, marriage status, smoking, and drinking habit. Power calculations were performed to ensure the adequate sample size. Pearson correlation analysis was used to compare the quantification results obtained using different methods. A *P* value of <0.05 was considered statistically significant, and all probabilities were two tailed. All calculations and image analyses were performed using in-house programs written in the R language.

## Results

Demographic and clinical characteristics of patients and healthy controls

A total of 65 patients with schizophrenia, 29 patients with mood disorders (included 18 patients with major depression and 11 with bipolar disorder) and 62 matched healthy controls were included in this study. Subjects with diabetes, malignant tumors, fever, inflammation, or other physical diseases were excluded. The demographics and clinical information for all the subjects are shown in supplementary Table S1. There was no significant difference between the three groups in terms of age, sex, body mass index (BMI), or smoking and drinking habits (Table S1).

### Increased cfDNA levels in schizophrenia patients

**Fig. 1:**
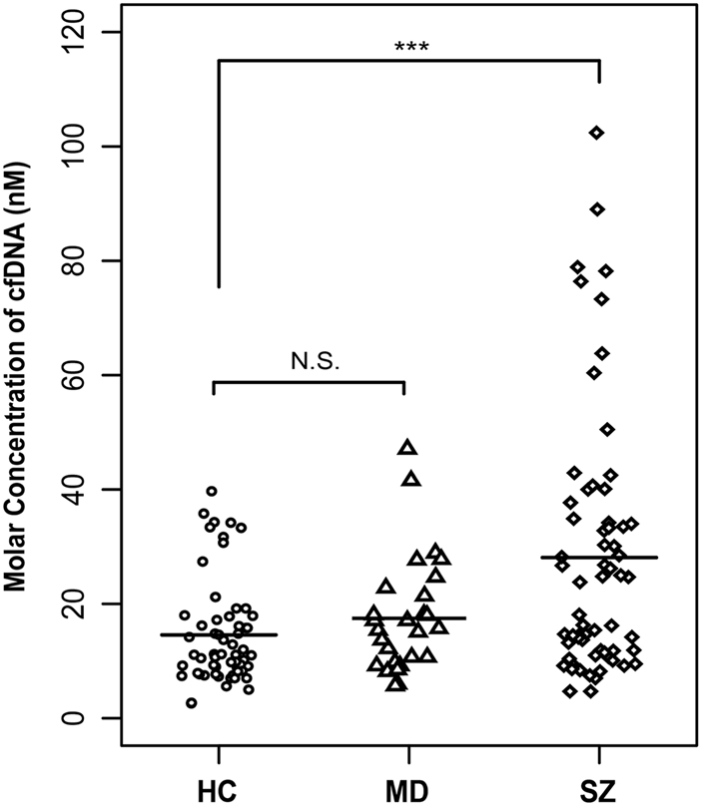
Molar concentrations of cfDNA in the three groups. Statistical comparisons were performed using logistic regression analysis. SZ schizophrenia, MD mood disorders, HC healthy controls, N.S. no significant difference, *** *P* < 0.001

The molar concentrations measured by FCS of cfDNA from all patients and healthy controls were analyzed using FCS. The levels of cfDNA in the SZ group were approximately two-fold higher than those in the HC group, whereas there was no significant difference between the cfDNA levels detected in the MD group and in the HC group (SZ group (29 ± 15) nM) vs. HC group ((15 ± 9) nM, *P-*value = 0.00062); MD group ((17 ± 10) nM) vs. HC group ((15 ± 9) nM, *P*-value = 0.343; Fig. 1).

### Size distribution analysis of cfDNA

After data processing described in supplementary, we obtained the DNA distribution data for 96 diffusing components; diffusion times ranged from 0.1 to 100 ms and one or two peaks (Figure S3) were detected for each sample. The diffusion times represented relative molecular sizes.

**Fig. 2:**
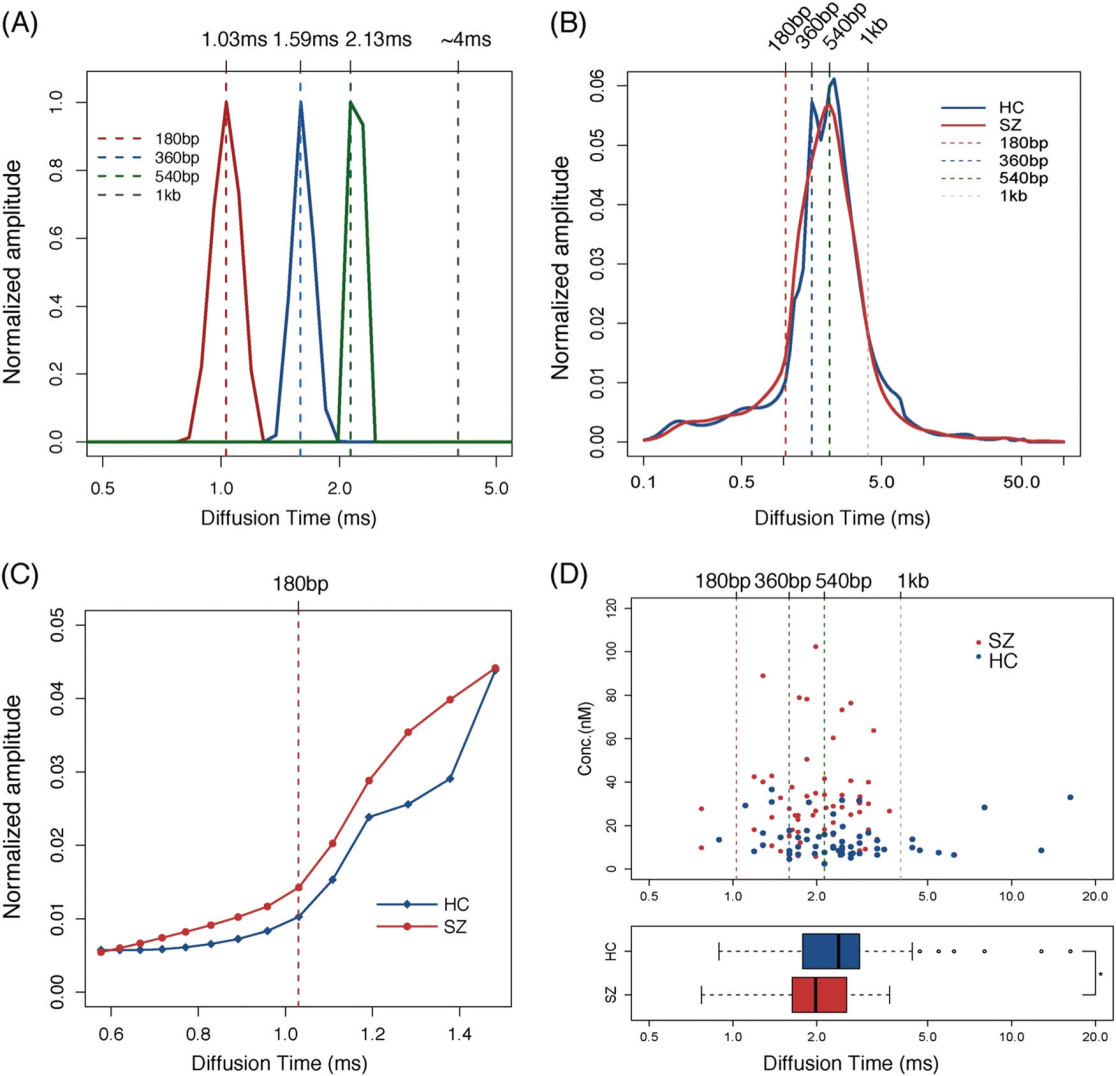
Characteristics of the size distributions of cfDNA in SZ and HC groups. **a** The solid lines are the distribution curves and the dotted lines indicate the diffusion times of the peak points of the markers. **b** The figure shows the average amplitude of each diffusion component in the schizophrenia group and the healthy control group. **c** The enlargement part of the diffusion components from 0.6 to 1.4 ms, which represents the scope with major differences in size. The dotted lines are the positions of the markers. **d** Average diffusion time of samples. Every dot represents the average diffusion time of each sample in the upper figure. The figure below is the boxplot of the average diffusion time of each sample in the SZ and HC group. SZ schizophrenia, HC healthy controls; * *P* *<* 0.05

The molecular size of cfDNA was determined using three DNA base pairs with lengths of 180 bp, 360 bp, and 540 bp, which represent mono, di, and tri-nucleosomal lengths, respectively. The corresponding diffusion times of the three base pairs are 1.03,1.59, and 2.13 ms, respectively (Fig. 2a). A 1000 bp DNA base pairs (which we used in our previous study) is also shown in Fig. 2a.

The average amplitude of each component was calculated for both the SZ group and the HC group. Figure 2b shows that the diffusion times for the majority of DNA molecules ranged from 1 to 4 ms, corresponding to a size of ~180 bp to 1 kb. The figure also shows that the size distributions for the SZ group cfDNA were shifted slightly toward the shorter end of the range compared with the HC group cfDNA. Figure 2c shows that the cfDNA amplitude in the SZ group was higher than that in the HC group for diffusion times ranging from 0.6 to 1.5 ms, which corresponds to DNA molecules shorter than 360 bp, suggesting that there is a higher concentration of short DNA molecules in the SZ group.

We calculated the average diffusion time as a representative value for the average cfDNA length for each sample. The calculation methods were also described in supplementary. All the samples with an average cfDNA length longer than 1 kb appeared only in the HC group, and samples with that shorter than 540 bp were clearly present at a higher concentration in the SZ group than in the HC group (Fig. 2d). In addition, the average diffusion time in the SZ group was significantly decreased compared with that in the HC group (The boxplot in Fig. 2d, *P*-value = 0.028). The results further showed that the increased amount of short DNA molecules in the SZ group accounted for most of the variation in cfDNA concentration across groups.

### Validation of results using fluorometry and qPCR

**Fig. 3:**
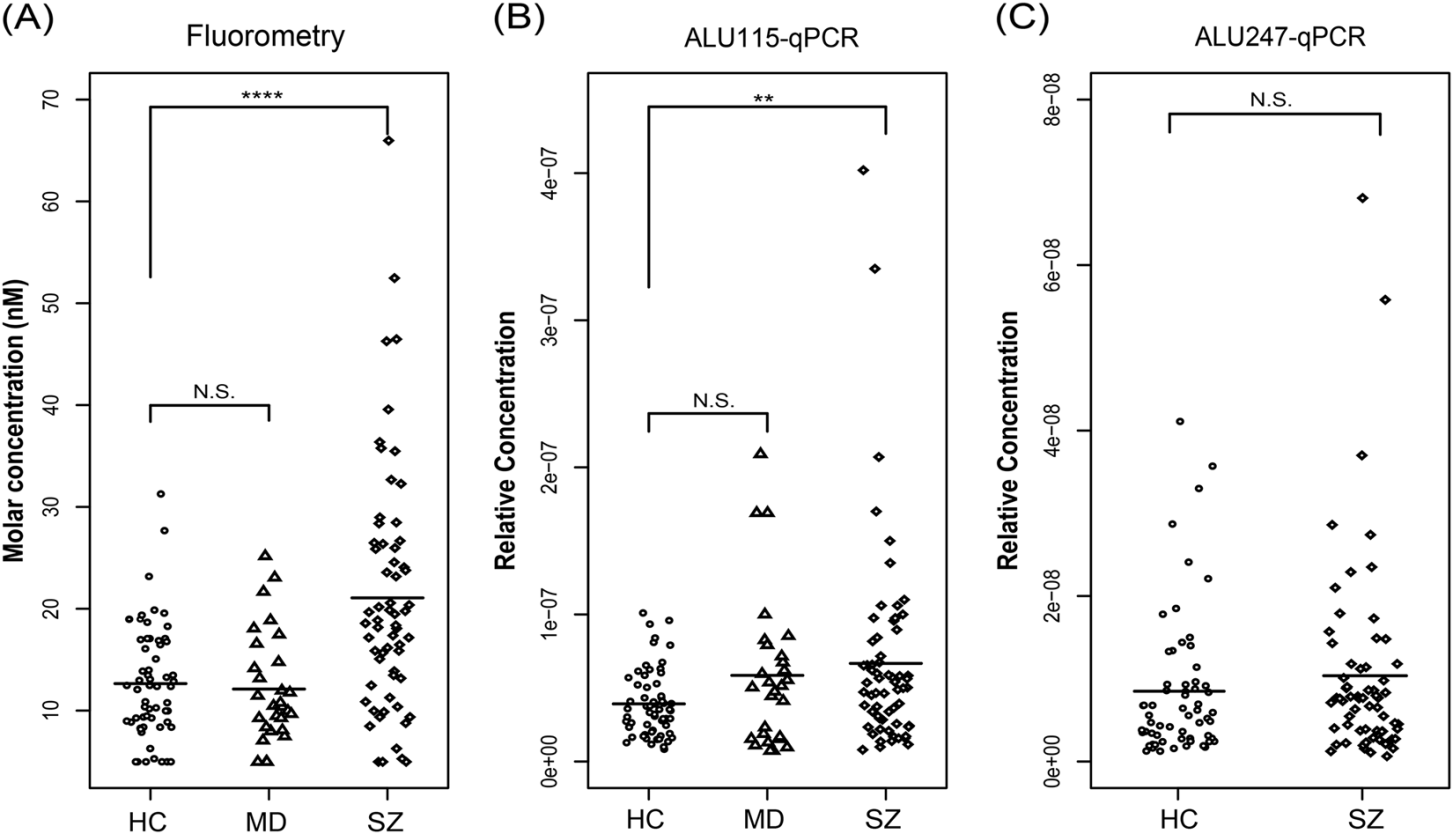
cfDNA levels measured by Fluorometry and qPCR. Statistical comparisons were performed using logistic regression analysis. SZ schizophrenia, MD mood disorders, HC healthy controls, N.S. no significant difference, ** *P* *<* 0.01; ***P *<* 0.001

To verify the results obtained by FCS, we further detected cfDNA using a QubitVR 2.0 Fluorometer and qPCR. Consistently, the mass concentration of cfDNA was significantly increased in the SZ group compared with in the HC group (SZ group: (21.1 ± 12.0) ng/mL; HC group: (12.7 ± 5.5) ng/mL; *P*-value = 0.000081), whereas the mass concentration of cfDNA in the MD group was not significantly different from that in the HC group (MD group: (12.2 ± 5.4) ng/mL; HC group: (12.7 ± 5.5) ng/mL; *P*-value = 0.443). The same results were also obtained using qPCR of 115 bp amplicons (SZ group (6.7e-08 ± 6.8e-08) vs. HC group (3.9e-08 ± 2.3e-08) *P*-value = 0.0089; MD group (5.9e-08 ± 5.1e-08) vs. HC group (3.9e-08 ± 2.3e-08) *P*-value = 0.056, Fig. 3). Moreover, there was no difference between the SZ group and the HC group in the levels of long-fragment DNA amplified by 247 bp ALU amplicons (SZ: 1.0e-08 ± 1.2e-08; HC: 0.9e-08 ± 0.9e-08; *P*-value = 0.506), suggesting that only short DNA molecules were present at a higher concentration in the SZ group (Fig. 3).

**Fig. 4:**
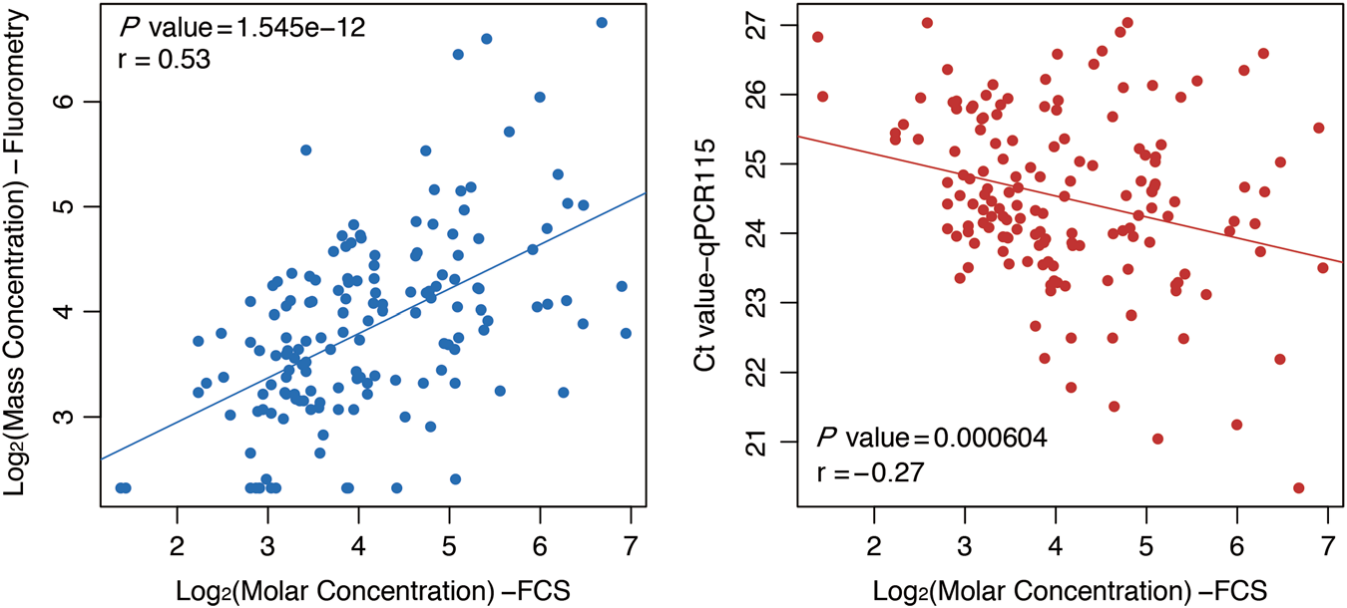
Correlations between cfDNA concentrations measured by the three methods. Scatterplot comparing the correlations between molar concentrations measured by FCS and mass concentration measured by fluorometry (red) and Ct values by qPCR (blue). The correlation coefficient and the *P* value was calculated using Pearson’s correlation analysis

We further analyzed the correlations of the quantification results from FCS with those from qPCR and those from fluorometry using Pearson’s correlation coefficient and trend chart (Fig. 4). We found a significant positive correlation between the molar concentrations measured by FCS with mass concentrations measured by fluorometry, and a significant negative correlation between the molar concentration with the Ct values obtained by qPCR. These results further validated the above conclusions that levels of cfDNA were significantly increased in schizophrenia patients and exhibited a shorter size distribution pattern.

## Discussion

In this study, we investigated the characteristics of cfDNA in patients with schizophrenia, patients with mood disorders, and matched healthy controls using FCS. First, cfDNA levels were approximately two-fold higher in schizophrenia patients than in healthy controls, but levels in patients with mood disorders were not significantly different from those in healthy controls (Figs. 1 and 3). Second, compared with cfDNA from healthy controls, cfDNA from schizophrenia patients comprised an increased concentration of short DNA molecules and showed an apoptosis-like size distribution pattern (Figs. 2 and 3).

Since the first observation of cfDNA, it has been detected in many diseases using multiple methods such as electrophoresis, fluorometry, qPCR, microscopy and massively parallel sequencing. Here, we analyzed the concentrations and size characteristics of cfDNA in schizophrenia patients using a new method called FCS, and the results regarding both of these aspects were verified by qPCR and fluorometric quantification (Figs. 3 and 4). The apoptosis-like size distribution pattern of cfDNA in schizophrenia patients is in agreement with what has been observed by massively parallel sequencing in other diseases (Fig. 2). qPCR and massively parallel sequencing have been widely used to analyze the size distributions of cfDNA in tumors, pregnancy, and some other conditions, but limitations remain. For example, qPCR is only applicable to preselected loci, and so it cannot provide a complete and systematic analysis of cfDNA. Although massively parallel sequencing is a powerful method that can provide abundant information and contribute to the understanding of disease, it is time consuming, sample intensive, and costly. FCS is a quick and easy method for cfDNA detection in which only 2.5 ng DNA and 240 s are needed for each sample. The relative concentration of DNA fragments >1000 bp can also be calculated, which is beyond the detection limits of both qPCR and massively parallel sequencing. Direct measurement of plasma DNA without extraction could be accomplished in the future through technique optimization, which will provide a more convenient method to help cfDNA to serve as an effective biomarker in clinical practice.

Accumulation of cfDNA in plasma or serum results from an excessive release of DNA following massive cell death. Fragments of ~180 bp (or multiples of 180 bp) are frequently derived from apoptotic cells, whereas fragments larger than ~10,000 bp are from cells undergoing necrosis^12,18^. The increased levels of cfDNA observed in this study suggest that the levels of fragmented DNA were significantly increased in the plasma of schizophrenia patients compared with in the plasma of healthy controls. In terms of size analysis, cfDNA mostly contained short fragments (<1000 bp) and there was an increased proportion of apoptotsis-specific fragments (from mono to trinucleosomal DNA fragments) in schizophrenia patients compared with in healthy controls (Fig. 1). These results indicate that apoptosis is activated in the acute stages of schizophrenia and that it may play roles in the pathophysiology of the disease. Early studies examining the potential role of apoptosis in schizophrenia have reported aberrations in apoptosis-regulating proteins and DNA fragmentation status in the postmortem temporal cortex^4,24^. As previous studies have demonstrated decreased Bcl-2 levels, increased Bax/Bcl-2 ratio and unaltered Caspase-3, it was proposed that increased vulnerability to pro-apoptotic stimuli occurred in the early stages of schizophrenia, but not in chronic schizophrenia. In addition, early results did not assess specific markers of apoptotic cells, it was considered that large-scale cell loss does not occur in schizophrenia^25^. However, the increased cfDNA levels in the plasma of schizophrenia patients and the cfDNA size characteristics observed in this study could provide direct evidence supporting that cell apoptosis is increased in the pathogenesis of schizophrenia.

**Fig. 5:**
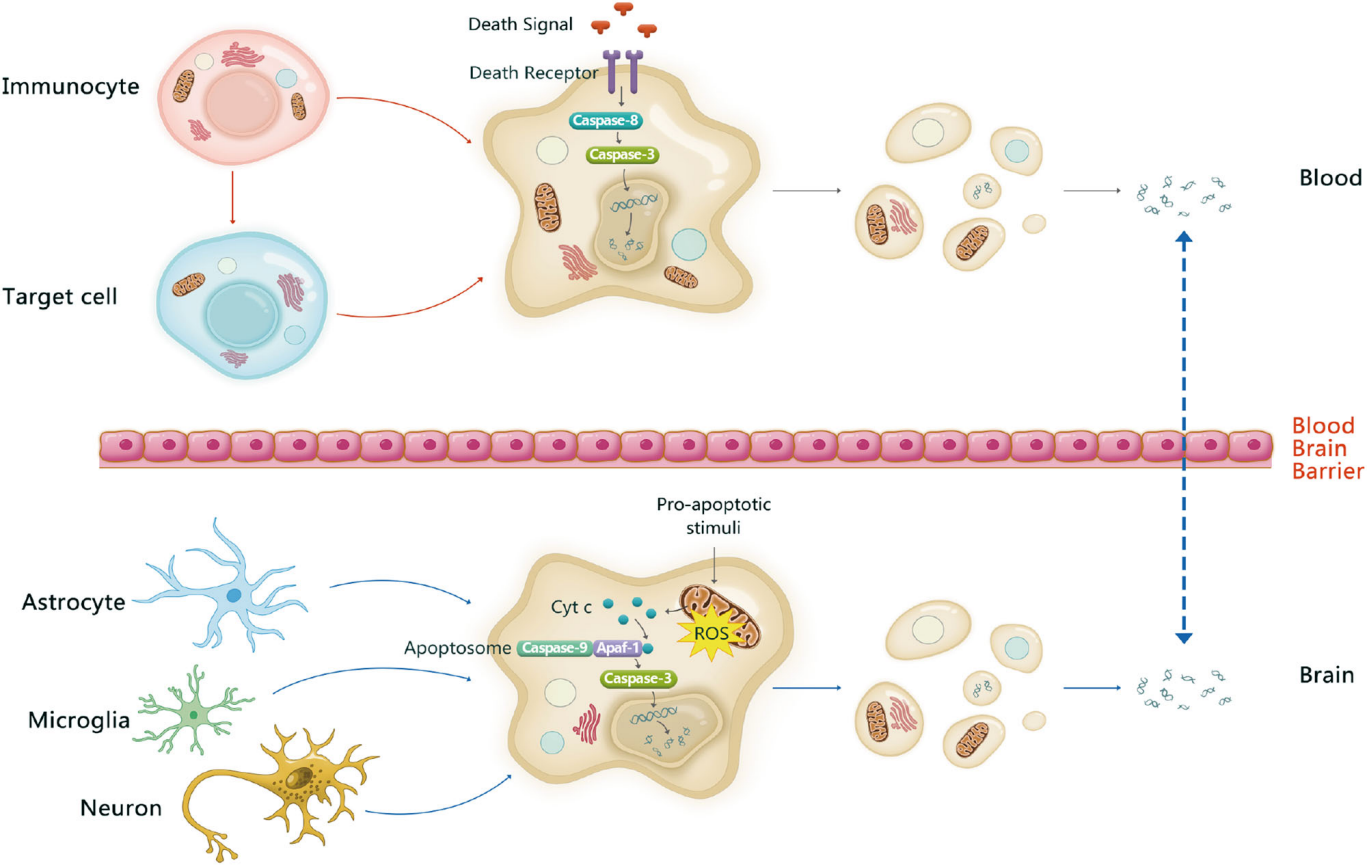
Possible origins of cfDNA in schizophrenia. In brain, pro-apoptotic stimuli may lead to the accumulation of ROS, the release of Cyt c and further trigger neural cells and neurogliocytes to undergo apoptosis. Fragmented DNA from apoptotic cells is released into the cerebrospinal fluid and flows into the blood through the blood–brain barrier. On the other hand, immunocytes in blood can either be induced to undergo apoptosis by a death signal or induce target cells to undergo apoptosis during an immune response. So the cfDNA observed in the plasma may come from the brain and the blood

Based on the above opinions, it is interesting to explore where the apoptosis takes place and define the underlying activation mechanism. Recently, researchers have developed a method to infer the contributing cell types by analyzing the “footprints” of protein-DNA interactions using deep sequencing. They found that cfDNA was primarily derived from myeloid and lymphoid cell lineages in healthy individuals, whereas contributions from some other tissues may be present in certain medical or physiological conditions^26^. Many dysfunctions documented in schizophrenia—such as oxidative damage, altered inflammatory cytokines, and abnormal dopamine or glutamate signaling—can induce apoptosis directly or through increasing cytochrome c release^27^. These pro-apoptotic stimuli may lead to the accumulation of ROS in mitochondria and further trigger neural cells and neurogliocytes to undergo apoptosis. Fragmented DNA from these cells may be released into the cerebrospinal fluid and flow into the blood through the blood–brain barrier (Fig. 5). Except for the activated apoptosis occurred in the brain, evidence from epidemiological, genetic and peripheral biomarker studies of schizophrenia all suggest the dysfunction of immune system, where apoptosis plays important roles. Therefore, we suspect that increased amounts of cfDNA were partially released from immunocytes in peripheral blood. Immunocytes can either be induced to undergo apoptosis themselves by a death signal or induce target cells to undergo apoptosis during an immune response (Fig. 5). In further studies, massively parallel sequencing could be performed to explore this conjecture and provide insight into the pathogenesis of schizophrenia.

As shown using three different methods, it is likely that cfDNA levels are increased only in subjects with schizophrenia and not in those with bipolar and major depression, suggesting that cfDNA aberrations may be specific to schizophrenia rather than common to all psychiatric diseases. It will be useful to explore the use of cfDNA as a biomarker for distinguishing schizophrenia from other psychiatric diseases in clinical diagnosis. However, further studies with larger sample sizes are needed.

In this study, we have provided a new method for detecting cfDNA that is more convenient and effective than existing methods, and using this method we have found that cfDNA in schizophrenia patients mainly originates from cell apoptosis. In addition, the elevated concentration and reduced size of cfDNA in schizophrenia patients (compared with cfDNA in patients with mood disorders and in healthy controls) suggests that levels of apoptosis are increased in schizophrenia. These features make cfDNA might be useful as an auxiliary biomarker for the diagnosis of schizophrenia. Based on previous hypotheses, we speculate that the origins of the cfDNA are immunocytes and/or neural cells. Further studies using massively parallel sequencing or larger sample sizes and specific fragment investigation may provide a more in-depth understanding of cfDNA in schizophrenia patients and the potential value of cfDNA in clinical practice.

## Electronic supplementary material

Supplementary
