# Supplementary material for: Analysis of the concentrations and size distributions of cell-free DNA in schizophrenia using fluorescence correlation spectroscopy
Source: Transl Psychiatry. 2018 May 22;8:104. doi: 10.1038/s41398-018-0153-3 (PMC5966419; doi:10.1038/s41398-018-0153-3)
Supplement: Supplementary file 1 — Supplementary [file 41398_2018_153_MOESM1_ESM.docx]

| Table S1: Demographics and clinical characteristics of patients and healthy controls | | | | | |
| --- | --- | --- | --- | --- | --- |
|  | **SZ**  **N=65** | **MD**  **N=29** | **HC**  **N=62** | ***P* value (SZ/HC)** | ***P* value (MD/HC)** |
| Age in years, mean (SD) | 37(11) | 38(14) | 34(11) | 0.109^a^ | 0.092^a^ |
| Sex(male/female) | 39/26 | 10/19 | 28/34 | 0.111^b^ | 0.370^b^ |
| BMI, mean (SD) | 21.6(3.8) | 21.6(3.9) | 21.7(2.3) | 0.847^a^ | 0.812^a^ |
| Marital status (N) | |  |  | 0.016^b^ | 0.166^b^ |
| Single | 28 | 6 | 17 |  |  |
| Married | 31 | 20 | 44 |  |  |
| Other | 6 | 3 | 1 |  |  |
| Smoke habit | |  |  | 0.139^b^ | 0.601^b^ |
| Smoker (N) | 20 | 7 | 12 |  |  |
| Non-smoker (N) | 45 | 22 | 50 |  |  |
| Course of disease | |  |  |  |  |
| First-episode (N) | 42 | 20 | - |  |  |
| Relapsed (N) | 23 | 9 | - |  |  |
| Family history | |  |  |  |  |
| Yes (N) | 15 | 5 | - |  |  |
| No (N) | 50 | 24 | - |  |  |
| SZ: schizophrenia, MD: mood disorders, HC: healthy controls, a: Student t-test, b: χ2 test  Table S2: Primers used in the study | | | | | |

Supplementary Information

| Amplicon | Forward primer (5'->3') | Reverse primer(5'->3') |
| --- | --- | --- |
| 180 bp | TTGAGGCTATCCAGGGGAAT | ACACACATTAGAACGCTGGA |
| 360 bp | TGCTGGGAAACAGGATTCAG | CACACACATTAGAACGCTGG |
| 540 bp | CACCACTTATGTGTTGCCTG | ATCCTGTTTCCCAGCAAGATG |
| ALU115 bp | CCTGAGGTCAGGAGTTCGAG | CCCGAGTAGCTGGGATTACA |
| ALU247 bp | GTGGCTCACGCCTGTAATC | CAGGCTGGAGTGCAGTGG |

Procedures of FCS assay

**Sample Preparation**

1. cfDNA isolated from 0.6ml plasma were dissolved in 30 μL ddH2O as descripted in the main body. 5 μL of cfDNA solution was 4-fold diluted using 20 mM Tris-HCL buffer (pH 7.4) for each sample.

2. SYBR Green I (10,000 X concentrate in DMSO) purchased from Molecular Probes Inc. (S7563, 796325, USA) was 20000-fold diluted using 20 mM Tris-HCL buffer (pH 7.4) to obtain a 0.5 X SYBR Green I solution.

3. Final detecting solutions for FCS measurement were prepared by mixing the equal volumes of diluted DNA with 0.5 X SYBR Green I. 20 μL final solution were used for FCS assay.

**FCS Detection and Data processing**

1. **FCS instrument and corresponding theory**

All FCS experiments were carried out on a FCS instrument constructed on an inverted fluorescence microscope (IX 71, Olympus, Japan). The excitation source was a 488 nm Sapphire LP USB CDRH laser (Coherent, USA). The excitation power can be controlled by a circular neutral density filter. The expanded laser beam was aligned and reflected into a water immersion objective (UplanApo, 60 X NA 1.2, Olympus, Japan) by a dichroic mirror (505DRLP, Omega Optical, USA). The excited fluorescence signal collected by the same objective, passed through the dichroic mirror (505DRLP, Omega Optical, USA) and then was filtered by a band-pass filter (530DF30, Omega Optical, USA) to block scattering light. Finally, the fluorescence was passed through a 35-µm diameter pinhole at the image plane in the front of single photon counting module (SPCM-AQR16, Perkin-Elmer EG&G, Canada). The temporal resolution of detector-SPCM is 350 ps. The fluorescence fluctuations were correlated with a digital correlator (Flex02–12D/C, Correlator. com, USA).

In FCS, fluorescence fluctuations of $\delta F\left(t\right)$ around the average fluorescenc$\left\langle F\right\rangle$were recorded in real time, and the autocorrelation curve from the fluorescence trace was calculated by a digital correlator as in eq. (1). The raw FCS data was nonlinearly fitted with Microcal Origin software package based on the Levenberg–Marquardt algorithm with eq. (2).

$G\left(\tau\right)=\frac{\left\langle\delta F\left(t\right)\times\delta F\left(t+\tau\right)\right\rangle}{\left\langle F\left(t\right)\right\rangle^{2}}, where \delta F\left(t\right)=F\left(t\right)-\left\langle F\left(t\right)\right\rangle$ (1)

$G(\tau)=\frac{1}{N}\times\frac{Te^{-\tau/\tau_{\text{r}}}}{1-T}\times\frac{1}{\left(1+\frac{\tau}{\tau_{D}}\right)}\times\frac{1}{\sqrt{1+(\frac{\omega_{0}}{z_{0}})^{2}\times\frac{\tau}{\tau_{D}}}}$ (2)

Here, τ is the decay time, N is the average number of fluorescent molecules in the detection volume, which is the reciprocal of G (0); T and τr are the fractional population and decay time of the triplet state for organic dyes or be related with chemical equilibrium such as the interaction of SYBR green I with DNA, τD is the characteristic diffusion time, and ω0 and z0 are the effective half radial and axial dimensions of the focal volume, respectively.

1. **Instrument calibration**

8 nM Rhodamine Green dissolved in water, which was also purchased from Molecular Probes Inc., was used as a reference dye for instrument calibration and concentration measurement, and the detection volume size was determined to be about 0.5 fL.

1. **Sample measurement**

20 μL final solutions were placed on the cover glass for FCS experiments. It was analyzed over a period of 240 s at room temperature (about 25 °C) in a single run, and was repeated 3 times.

1. **Data processing of FCS assay**

**(1) Concentration calculation**

The concentration of cfDNA (C_cfDNA_) was determined with FCS method (as described in eq. (3)) by comparing the N value of cfDNA with that of Rhodamine green with known concentration. After collecting fluorescence data for 240s, autocorrelation curves of cfDNA and Rhodamine green were obtained as presented in Figure S1 and Figure S2. Fitting these raw curves with eq. (2), the N values of cfDNA and Rhodamine green was determined. The concentration of Rhodamine green (C_RG_) was determined with the UV-Vis absorbance spectroscopy based on its molar extinction coefficients. The average of three repeated values was used to determine the final concentrations for each sample.

$C_{cfDNA}=\frac{N_{cfDNA}}{N_{RG}}\times C_{RG}$ (3)


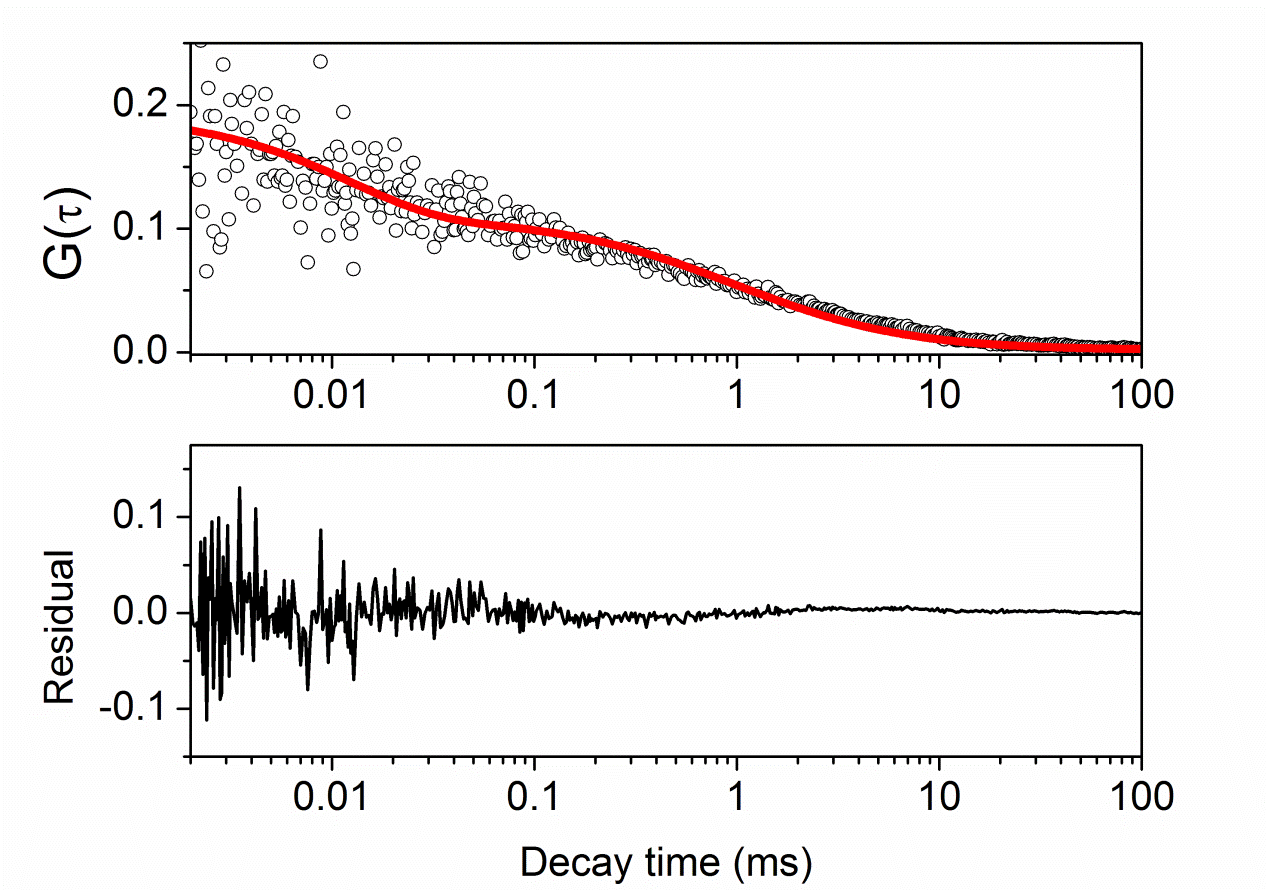

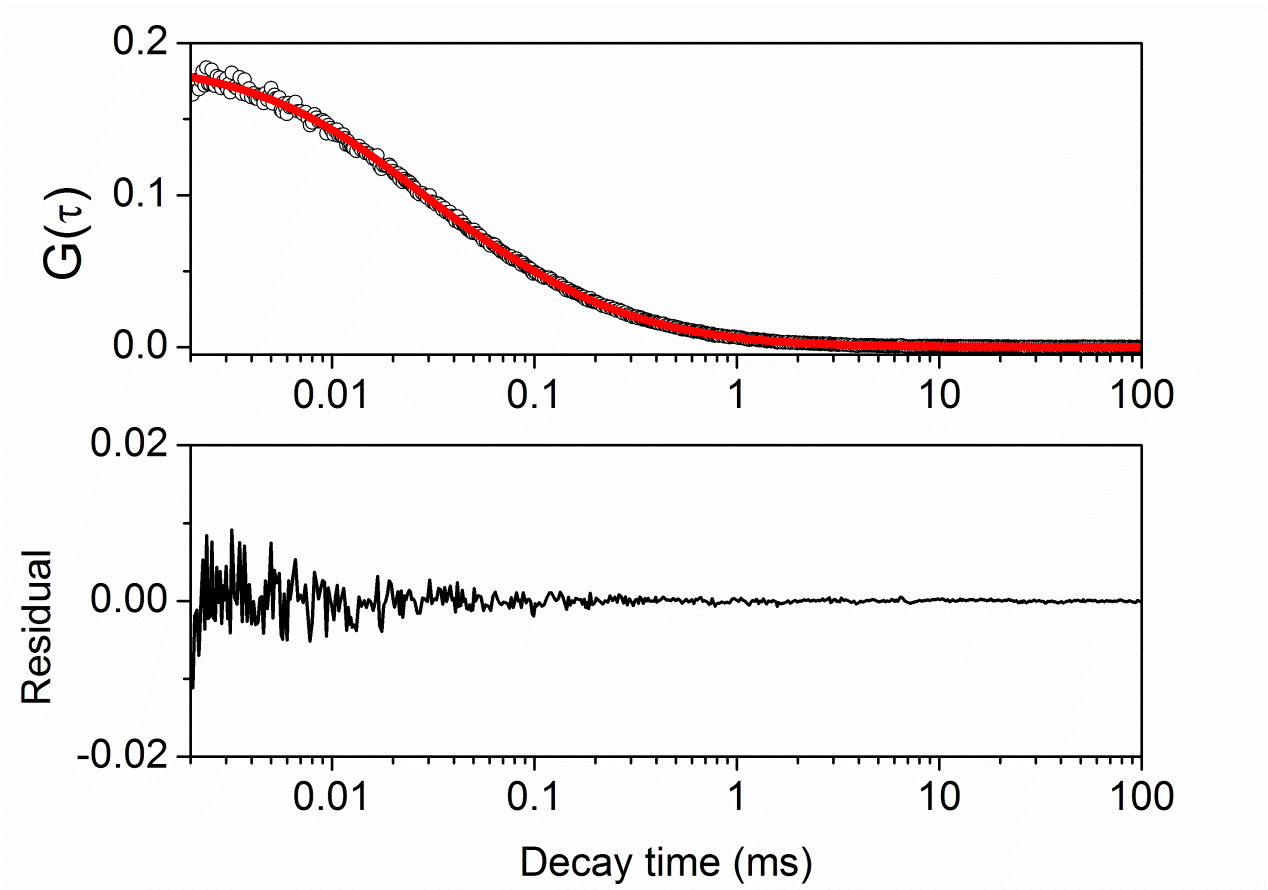


Figure S1. The typical raw FCS curves of cfDNA and Rhodamine green (circle), their fitting curves (red solid line) and their residual curves.

cfDNA

Rhodamine green

In order to demonstrate the low interference of SYBR Green I itself on the signal from dsDNA-dye-complex, additional experiments were performed. As shown in the following Figure S2, only if cfDNA combined with SYBR Green I, the signal could be detected (6-7kHZ). When cfDNA and dye existed in the solution separately, the signal was almost below the detection threshold. The autocorrelation curve of the sample in the presence of SYBR Green I could be fitted with FCS theoretical model (Figure S1), while the autocorrelation curves of 0.25 X SYBR Green I alone and cfDNA sample alone cannot be fitted (Figure S3).


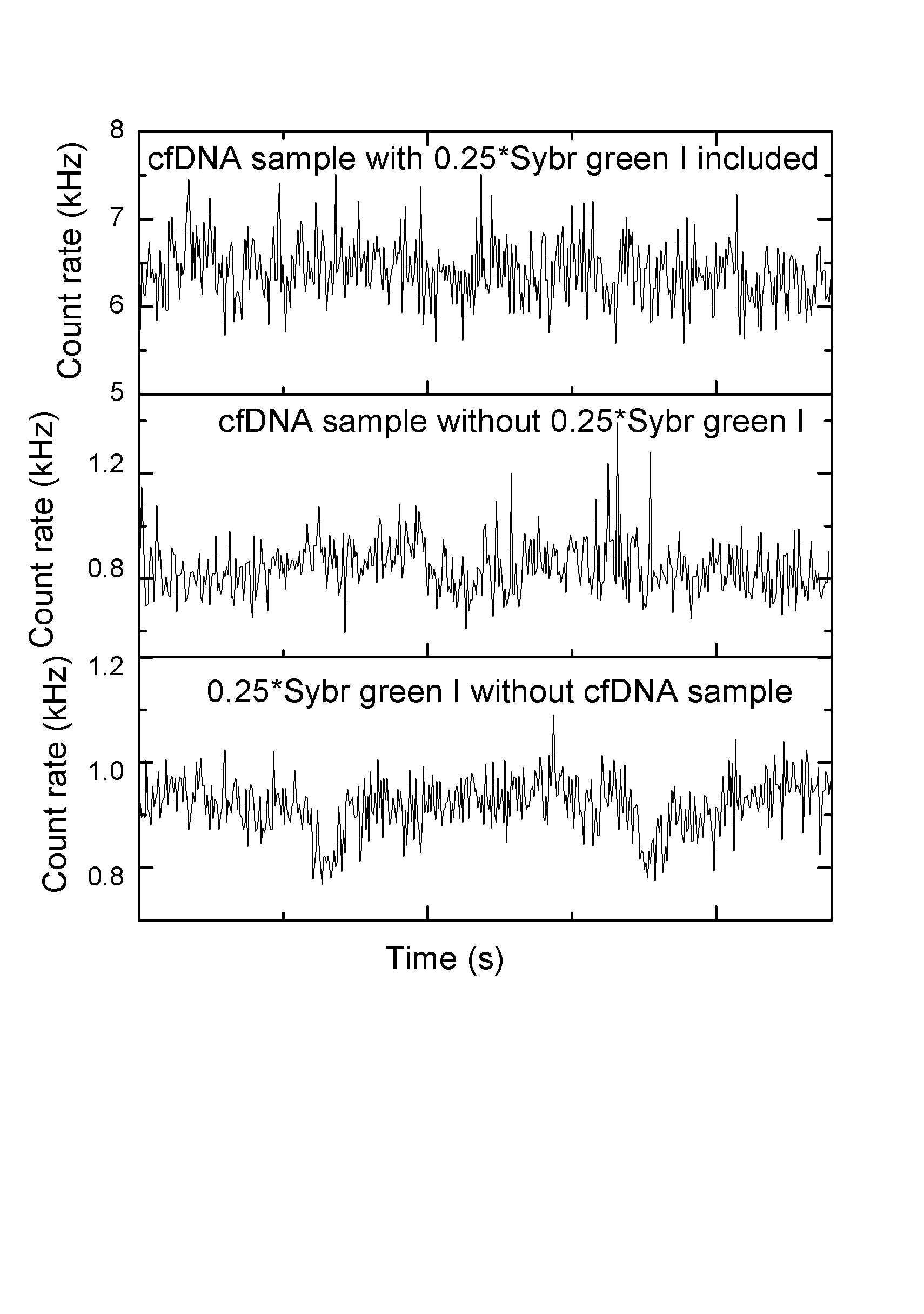


Figure S2. The count rate traces of cfDNA sample with SYBR Green I, cfDNA sample alone, and SYBR Green I alone.


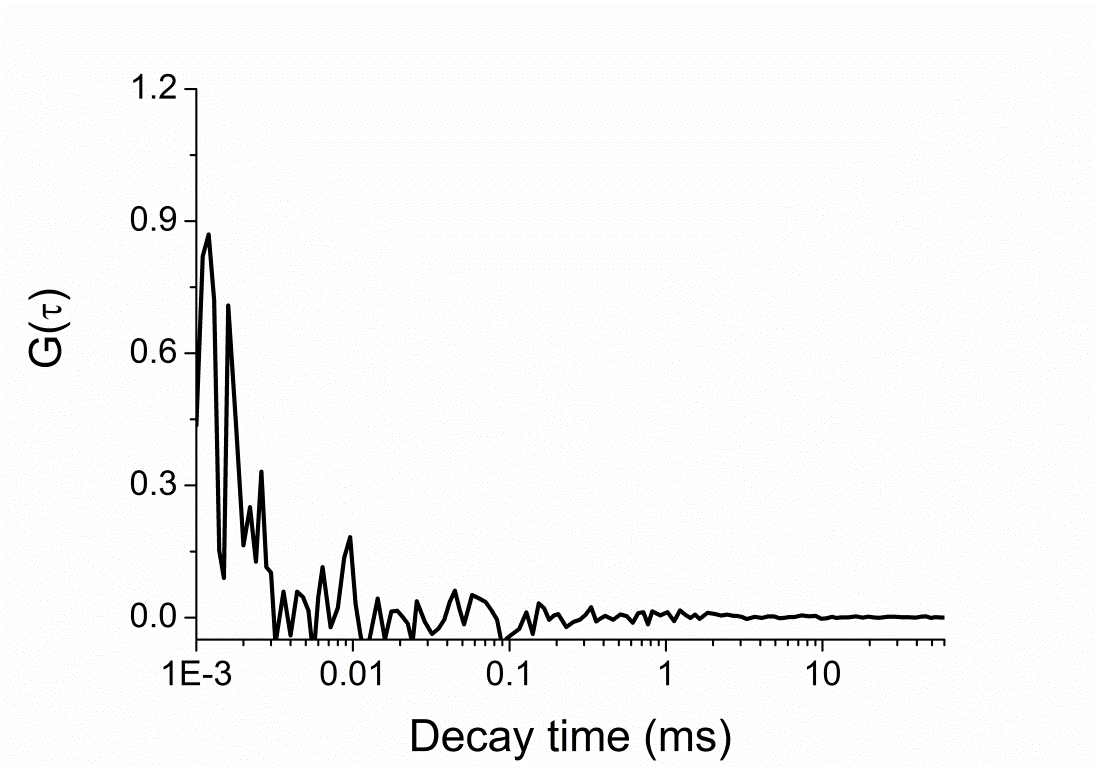

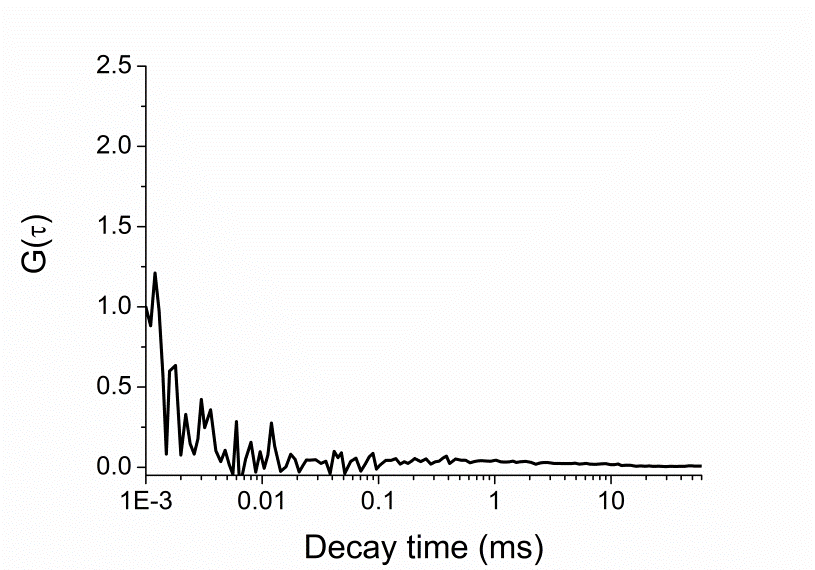


0.25 X SYBR Green I

cfDNA

Figure S3. Fitting curves of 0.25 X SYBR Green I without cfDNA sample and cfDNA sample without 0.25 X SYBR Green I

**(2) Size distribution analysis**

**Acquisition of diffusion time distribution curves**

FCS is a single molecule detection method using statistical analysis of the fluctuations of the fluorescence due to Brownian motion of fluorescent molecules. In the solutions, longer DNA fragments move around slower than the shorter ones, thus when combining with SYBR Green I longer DNAs has larger diffusion times in FCS assay. The diffusion time distribution curves (as in Figure S4) of different diffusing components in the DNA solutions was obtained by fitting the autocorrelation curves using the MEMFCS program provided by Professor Maiti. In this fitting algorithm, the initial number of diffusing components was set at 150. The lower value and upper value of diffusion times were set as 0.002 ms and 100 ms, respectively. In the fitted results, the distribution curve of diffusion times less than 0.1ms was not related to the molecular diffusion but, rather, was more attributable to the laser-induced photochemical reaction. A total of 96 diffusing components remained after deleting the times shorter than 0.1 ms.

The amplitude in distribution curves was normalized using the total amplitude of the 96 diffusing components in both the SZ group and the HC group. Peaks within the fitting curves were saved only if the peak height was one-third taller than the main peak height.


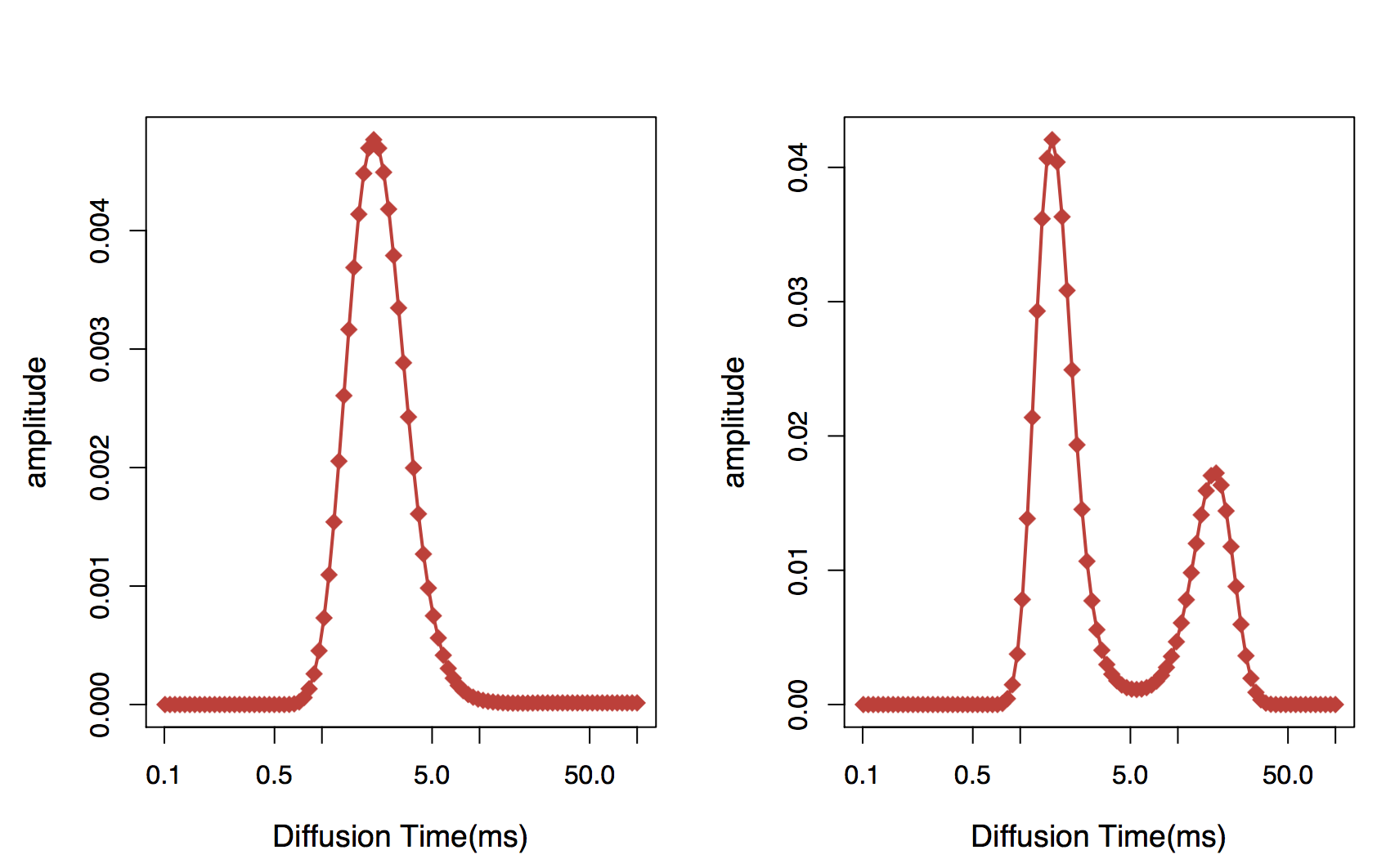


Figure S4. The typical distribution curves of cfDNA obtained by fitting the autocorrelation curves before normalization.

Here, we used three DNA base pairs with lengths of 180 bp, 360 bp, and 540 bp, which represent mono-, di-, and tri-nucleosomal lengths, respectively. The temporal autocorrelation curves are of 180 bp, 360 bp and 540 bp DNA are shown in Figure S5.

**Calculation of average diffusion times**

We calculated the average diffusion time as a representative value for the average cfDNA length for each sample. The calculation methods were as follows: the average diffusion time was the corresponding diffusion time of the main peak if there was only one peak, whereas it was the weighted average when two peaks were present (shown in Figure S4); weighting was based on the relative heights of the two peaks.


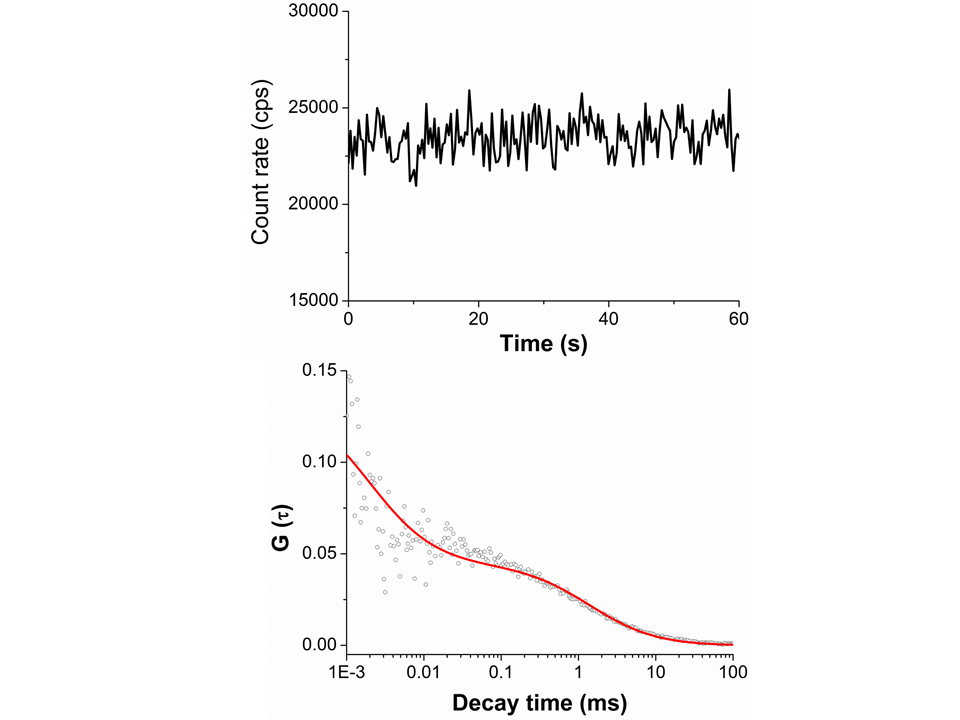

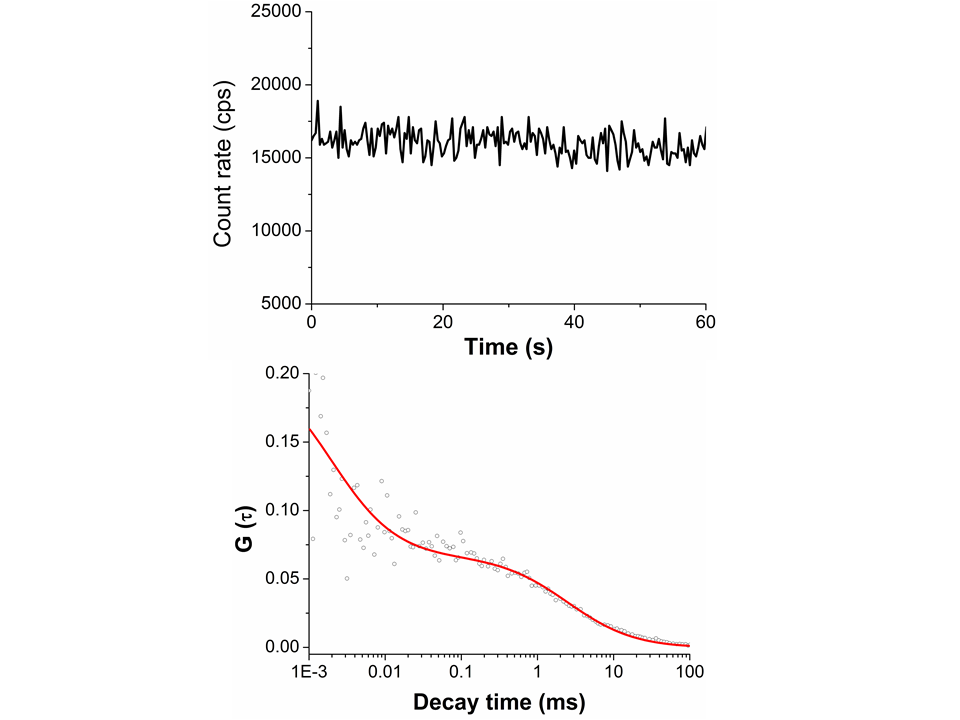

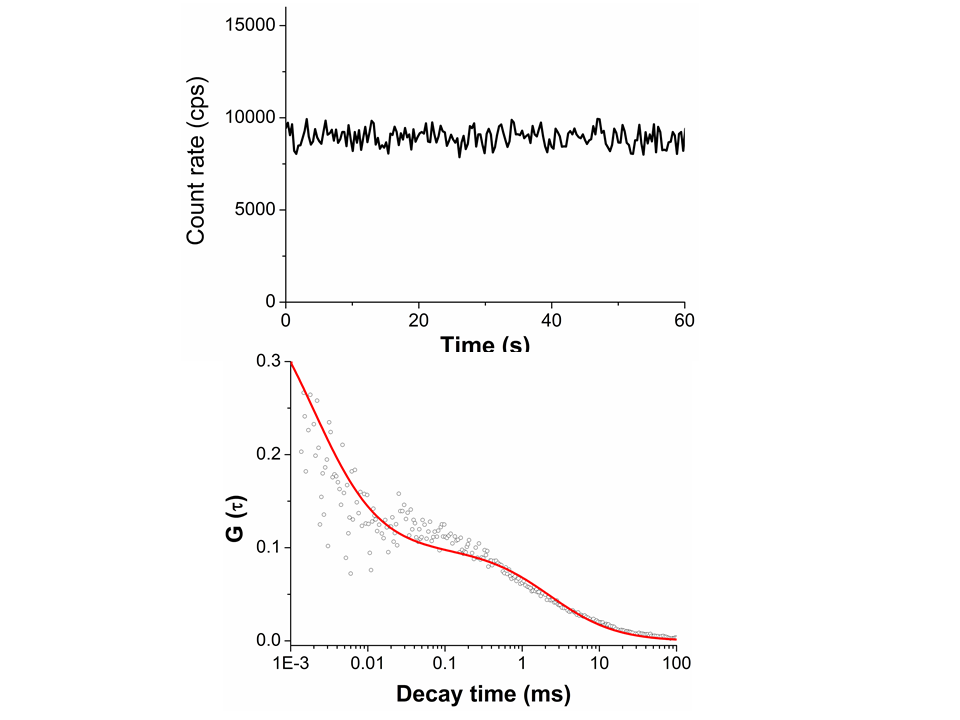


180 bp

360 bp

540 bp

Figure S5. The count rate trace (top) and the temporal autocorrelation curve (bottom) of 180 bp, 360 bp, and 540 bp DNA with SYBR Green I.
